# Accuracy of Trajectory Tracking Based on Nonlinear Guidance Logic for Hydrographic Unmanned Surface Vessels

**DOI:** 10.3390/s20030832

**Published:** 2020-02-04

**Authors:** Andrzej Stateczny, Pawel Burdziakowski, Klaudia Najdecka, Beata Domagalska-Stateczna

**Affiliations:** 1 Department of Geodesy, Faculty of Civil and Environmental Engineering, Gdansk University of Technology, Narutowicza 11-12, 80-233 Gdansk, Poland; andrzej.stateczny@pg.edu.pl; 2 Marine Technology Ltd., Roszczynialskiego 4/6, 81-521 Gdynia, Poland; k.najdecka@marinetechnology.pl (K.N.); b.domagalska@marinetechnology.pl (B.D.-S.)

**Keywords:** trajectory tracking, unmanned surface vehicle, navigation, bathymetry, hydrographic survey

## Abstract

A new trend in recent years for hydrographic measurement in water bodies is the use of unmanned surface vehicles (USVs). In the process of navigation by USVs, it is particularly important to control position precisely on the measuring profile. Precise navigation with respect to the measuring profile avoids registration of redundant data and thus saves time and survey costs. This article addresses the issue of precise navigation of the hydrographic unit on the measuring profile with the use of a nonlinear adaptive autopilot. The results of experiments concerning hydrographic measurements performed in real conditions using an USV are discussed.

## 1. Introduction

The importance of hydrographic measurement in recent years has been growing constantly due to the increasing use of water transport, including movements in restricted areas. A particular challenge is shallow-water measurements near land or navigational obstacles where the use of larger hydrographic units is impracticable or not justified economically. An important aspect in this respect is the desire to shift loads from roads and motorways to waterways, which, by definition, are safer and transport is more ecologically friendly and economical.

The process of conducting hydrographic measurements in land-restricted waterways requires precise acquisition of the measurement profile, and a limit to the acquisition of redundant data on the measurement strip tabs in the case of multi-beam echosounder (MBES) measurements and excessive gaps between measurement profiles in the case of single-beam sonar measurements. The International Hydrographic Organization (IHO) does not define the accuracy of maintaining the measuring unit on the profile, but only determines the percentage of searching the bottom of the reservoir and the accuracy of determining the position of the measuring unit. For instance, in the case of the most restrictive special category, 100% coverage and an accuracy of 2 m with respect to determination of position with a 95% confidence level is required [1].

Traditionally, for manned hydrographic units, the helmsman follows the position of the unit on the profile on the monitor screen of the measurement system, adjusting the parameters of the unit’s movement to the current weather conditions. Many measurement systems used throughout the world provide the helmsman with an indicator which shows the current distance from the planned measurement profile. Continuous tracking of the position of the measuring unit on the profile requires a high degree of concentration and an appropriate response from the helmsman; this is a tedious and challenging task. It requires a relatively frequent change of the watch in the case of the helmsman controlling the hydrographic unit.

A disadvantage of manual control is also the quite low accuracy of maintaining the measuring unit on the profile and a moment of inattention or distraction may result in deviation from the measuring profile thus requiring repetition of registration on the incorrectly registered profile. For this reason, significant amounts of redundant data are recorded, which hamper the processing of the measurement data. The issue of achieving precise control of the measurement profile on a hydrographic unit can be solved by using an unmanned surface vehicle (USV) with an adaptive autopilot which realizes precise control of both course and speed.

Generally, unmanned mobile platforms, including surface, aerial, and ground vehicles have been increasingly employed for numerous operations. Currently, the unmanned platforms are widely used around the world, giving rise to many new research opportunities. In this context, various control schemes have been also developed to perform predefined tasks. Unmanned surface vessels suffer from many uncertainties and unknown external disturbances like winds, waves, currents, etc., which inevitably lead to a very challenging task [2] like accurate trajectory tracking within this complex navigation environment [3]. Due to this, various advanced control techniques and schemes for surface vehicles have been developed, and the following groups can be highlighted: backstepping technique [4,5,6,7], chaos control approach [8,9,10], fuzzy control [2,3,11,12], neural control [13,14,15,16,17,18], and finite-time control [19,20,21].

This paper considers the problems of precise control of the measurement profile on a hydrographic unit using a nonlinear adaptive autopilot originally designed for unmanned aerial vehicles. The results of experiments carried out under real conditions are presented.

## 2. Trajectory Tracking

Precise tracking of the trajectory during maneuvering is very important, especially for an autonomous multi-purpose water platform [22,23] engaged in hydrographic survey missions of restricted access areas like ports, embankments, anchorages, bays and lakes, and rivers. Generally, in such areas, maneuvering requires execution of precise, previously planned track information. Additionally, in any difficult navigational situation such as caused by recreational boats and other traffic, precise execution of the planned or preplanned track becomes crucial.

The evaluated track following method, termed guidance logic, was designed originally for aerial applications [24] and unmanned aerial vehicles (UAVs) and was successfully implemented in many UAV applications [24,25,26]. This approach is also used by small unmanned surface vessels [27,28,29]; however, the platforms applied in that research are significantly smaller than the platform used in the present work. Studies on autonomous navigation algorithms and navigation strategies have been reported [30,31,32] while interesting studies for an adaptive system for steering strategy are available [33,34,35]. Aspects on the safety of vehicle navigation have been discussed [36,37,38] while actual problems concerning control of trajectory tracking for marine vehicles have also been reported [2,3,4,5,6,7,8,9,10,11,12,13,14,15,16,17,18,19,20,21,39,40].

**Figure 1:**
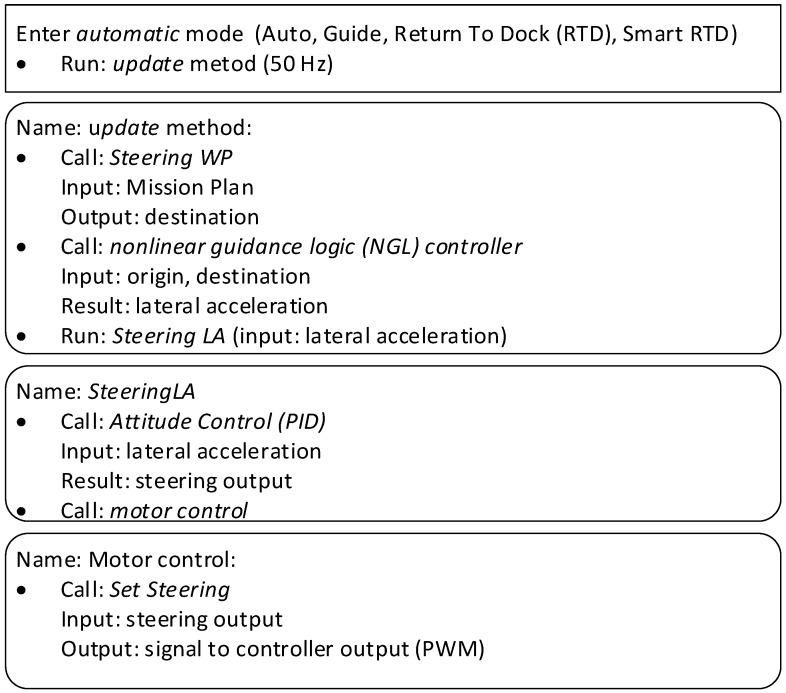
Trajectory tracking algorithm scheme.

An outline of the steps and the computer programing methods used for trajectory tracking is given in Figure 1. Implementation follows that of a previous study [41] and where the mission control module is similar to a system described elsewhere [22].

This algorithm requires a frequency of 50 Hz (method name: update), meaning that the update method is used 50 times per second. The method is activated only in automatic mode, meaning that only the automatic modes Auto, Guided, Return to Dock (RTD), and Smart RTD use this method. The automatic modes make use of all navigation sensors, and valid sensors readings are compulsory for automatic mode activation. Any missing sensors’ data are unable to activate this function. Moreover, the Auto mode requires a programmed route (route plan), the RTD mode requires a dock (home) position and a saved travelled route to perform smart RTD, and the Guided mode requires a desired point location. All data are provided in the form of geographical waypoints (WPs).

The update method uses, in order, the following methods:

- Steering WP—the basic route plan and vehicle destination. The method provides the location that the vehicle should achieve (destination).
- NLG controller—the method where the navigation process is implemented. The method, based on destination and vehicle origin, calculates the lateral acceleration.
- Steering LA—the method, based on the PID (proportional–integral–derivative) controller, calculates the steering output.
- Set Steering—the method converts the steering output to the appropriate navigation controller output signal. The output signal (PWM: Pulse-Width Modulation) is the electrical signal interpreted by the electric motor’s controller and the rudder’s linear actuator controller.

All the methods enumerated above participate in trajectory tracking and have been tuned separately during the platform development and validation process.

**Figure 2:**
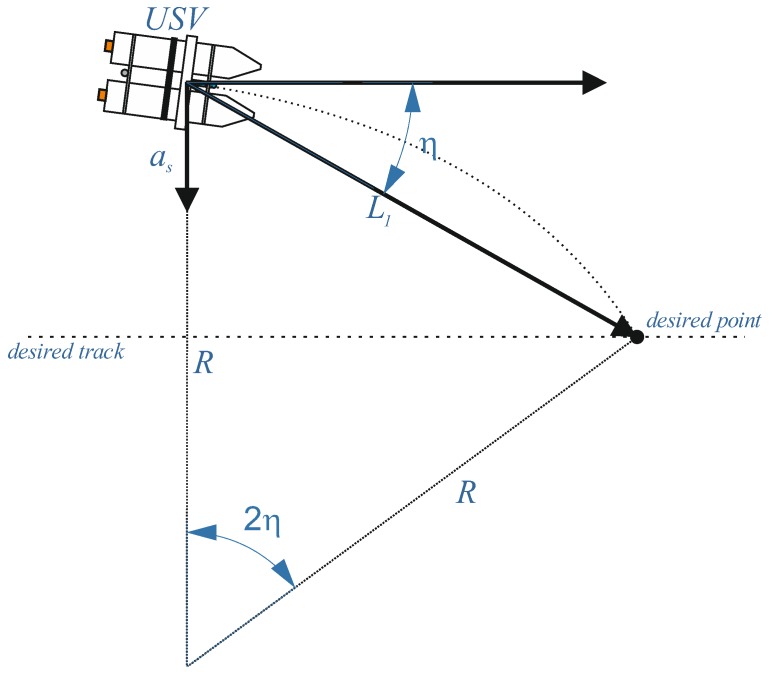
Guidance logic trajectory tracking.

The guidance logic [24] selects the desired point on the planned trajectory and generates a lateral acceleration ($a_{s})$ using this point, in accordance with following formula:

(1) $$a_{s}=2\frac{V^{2}}{L_{1}}\sin\eta$$

where the desired point is on the planned track at distance $L_{1}$ from the actual origin of the vehicle (Figure 2). Knowing that:

(2) $$L_{1}\;=\;2R\sin\eta$$

where the lateral acceleration calculated by Equation (1) equals the centripetal acceleration required to follow instantaneously a circular segment and $a_{s}$ is the property used to track a circle of any radius R. This characteristic predisposes the guidance logic to work faithfully with a curved path. Moreover, as shown elsewhere [24], the method shows better capabilities than the PID controller when used on the UAV in the presence of wind. In this research, the method will be examined on the unmanned surface vessel (USV) using state-of-the-art geodetic grade measurements. The reference position of the USV was measured using independently acquired GPS navigation data (GPS RTK (Real Time Kinematic) geodetic receiver).

The original Equations (1) and (2) were modified [41] for USV navigation purposes. The implementation required the addition of two parameters ($L_{1}$ Damping Factor and $L_{1}$ Period). Therefore, for the new $L_{1}$:

(3) $$L_{1t}=\frac{1}{\pi }\zeta Tv$$

ζ is the $L_{1}$ Damping Factor, T is the $L_{1}$ Period (s), and v is the speed of the unit. Finally, substituting Equation (3) into Equation (1) can be written as:

(4) $$a_{st}=\frac{k\;v^{2}}{L_{1t}}\sin(\eta_{1}-\eta_{2})$$

where k is the $L_{1}$ Control Gain, defined as:

(5) $$k=4\zeta^{2}$$

**Figure 3:**
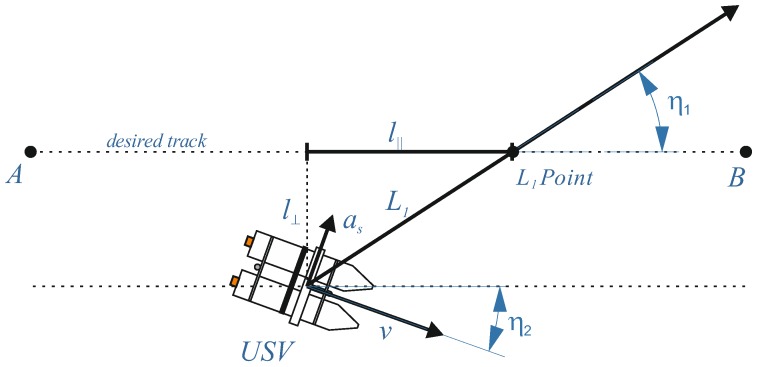
Guidance logic trajectory tracking for an unmanned surface vehicle (USV).

The cross-track error (XTE), corresponding to $l_{⊥}$ on Figure 3 and defined as a distance between actual USV position perpendicular to the intended (desired) track, can be approximated as a second order differential equation [42]:

(6) $$\ddot{d}+2\zeta \omega_{n}\dot{d}+\omega_{n}^{2}d\;=\;0$$

where the natural frequency $\omega_{n}$ (natural frequency (eigenfrequency) is the frequency at which a structure or system have the tendency to oscillate in the absence of any driving or damping forces), is related to T:

(7) $$\omega_{n}\;=\;\;\frac{2\pi }{T}$$

The dynamics of Equation (6) indicate that changing the values of ζ and $\omega_{n}$ adjusts the control response of XTE. The ζ and $\omega_{n}$ values are specified by the user and, for this research, were adjusted experimentally after long and extensive trials.

The parameter tuning requires some user experience regarding the USV’s parameters and the response observations during the trials. As a general rule for tuning, the following instructions are given. The $L_{1}$ period T is given in seconds with a range from 1 to 60 (increments of 1) for the $L_{1}$ tracking loop and is the primary control parameter for aggressive turns in auto mode (Figure 1). This parameter should be larger for less responsive USV platforms. For smaller and more maneuverable USVs a lesser value can be set. The starting value was adjusted experimentally as 20 s. The $L_{1}$ control damping ratio ζ with range from 0.6 to 1 (increments of 0.05) should be increased if the USV overshoots the track being followed.

Significant changes made to the original Equation (1) enable the length $L_{1}$ to be calculated dynamically by the navigation loop depending on the USV ground speed changes and enable the user to specify a constant period for the tracking loop.

The L1 Control Gain was changed from a fixed value of 2 (Equation (1)) to be calculated based on the ζ value set by the user. This enables additional damping to be specified to compensate for delays in the velocity measurement and for the USV frame to respond.

**Figure 4:**
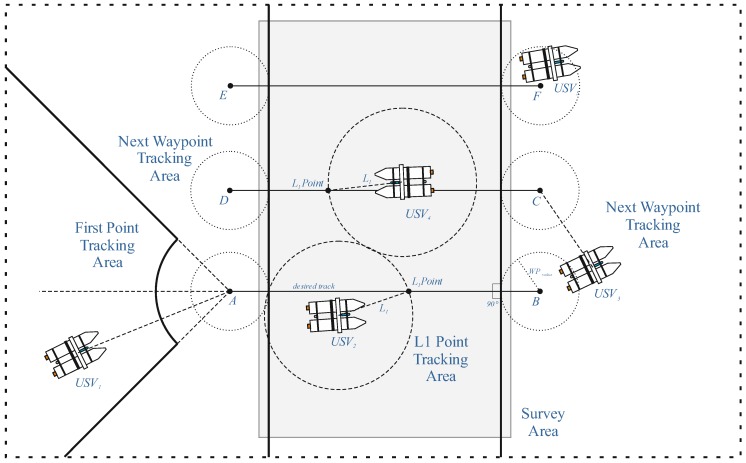
Tracking areas for USV hydrographic survey using L1 controller.

Figure 4 presents all possible tracking movements. The current tracking mode depends on the area where the USV is located in relation to the route plan. Figure 4 presents a simple survey plan based on 6 WPs. When the vehicle is located in $USV_{1}$, the first WP is acknowledged to be tracked and vehicle i proceeds to point A. The point is considered to be reached when the unit is within the $WP_{Radius}$ parameter. The $WP_{Radius}$ is the distance in meters from a WP when the algorithm considers the WP has been reached and determines when the unit will proceed to the next WP. After reaching the first WP, the unit starts the L1 tracking mode (positions $USV_{2}$, $USV_{4}$). In the L1 tracking mode a point L is tracked and this point is dynamically located on the line between the last and next WP. The L point is the intersection point between the track line and the circle with the radius equal to the distance $L_{1}$ defined by the user. When point B is reached the next WP (C) is acknowledged to be tracked (Next WP Tracking Area). The procedure is repeated until the unit reaches the last WP when it stops and waits for the next command, either from the operator or the autonomous system.

The L1 method calculates the lateral acceleration to be executed by the unit. The $L_{1}$ acceleration is translated to the motors and the steer command using the Steering LA and Motor Control methods (Figure 1). The Steering LA method is based on a PID controller. The automatic (mission control) algorithms, according to research [25] were demonstrated to control speed and course of the surface vessel. The PID controller continuously attempts to minimize the error e(t) over time by adjustment of a control variable u(t). The error value function e(t) is the difference between a desired set point r(t) and the measured process variable y(t) (e(t) = r(t)−y(t)). Process variable is represented by the value that is being controlled (e.g., actual speed or actual course). The PID controller can be expressed as:

(8) $$u\left(t\right)=K_{p}e\left(t\right)+\frac{1}{T_{i}}\overset{t}{\underset{0}{\int }}e\left(t\right)\mathrm{dt}+T_{d}\frac{\mathrm{de}\left(t\right)}{\mathrm{dt}}$$

where $K_{p}$, $K_{i}$, and $K_{d}$ are non-negative and denote the coefficients for the proportional, integral, and derivative terms, respectively. The parameters were set experimentally during test trials. The PID desired and the PID achieved were monitored and displayed and the coefficients can be adjusted to achieve the appropriate object response and parameter (course and speed) stabilization.

## 3. System Specification

**Figure 5:**
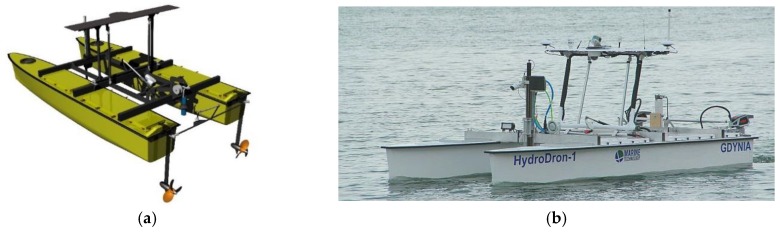
Combined skid and traditional steering system—motors and rudder design plan (**a**), photo of USV HydroDron during experiments (**b**).

The steering system specification for the unit (Table 1) is based on a combination of skid steering and a traditional rudder (Figure 5).

Skid-Steering is a type of vehicle steering where rotation (yaw) is obtained by a difference in the speeds of the left and right propellers (wheel) and is typical for vehicles with non-orientable propellers (wheels). Electric motors installed on the platform and situated in the right hull are turned using a linear actuator, therefore, maneuverability is enhanced, and the unit is more responsive to steering, when compared to steering using only pure skid steering. Additionally, this USV uses pivot turns when the angle of turn is greater than a specific angle (can be set in the Pivot Turn Angle parameter). The pivot turn angle was selected as a result of extensive trials and was equal to 45°. 

The USV is equipped with an autopilot and the navigation position is calculated based on the autopilot’s internal sensors: three magnetic compasses (nine MEMS magnetometers), an inertial navigation system (INS) based on simple MEMS (microelectromechanical system) sensors consisting of nine gyroscopes and nine accelerometers (compasses and INS embedded in autopilot) and an external GNSS receiver based on an UBlox M8N module. In that configuration, the USV position is calculated using an EKF (extended Kalman filter). The EKF is a 24-state extended Kalman filter and the autopilot’s filter estimates the following states: altitude, velocity, position, gyro bias offsets, gyro scale factors, Z accel. bias, Earth’s magnetic field, platform body magnetic field, and wind velocity. For calculations in this study, the navigation autopilot used the EKF output position, which means that this was not a pure GPS reading, but filtered and estimated based on other internal sensors. Only one GNSS module based on the UBlox M8N module was used (Table 2).

The GPS RTK module readings are not used for navigation; the module is part of the hydrographic equipment and additionally, the acquired RTK readings were used as a reference and as an independent USV position registration. The GPS RTK receiver specifications are given in Table 3. The GPS RTK used for position registration was a state-of-the-art survey grade receiver, embedded in the SPLITBOX-STD-T hydrographic equipment and based on the Trimble GNSS receiver.

## 4. Experiments

**Figure 6:**
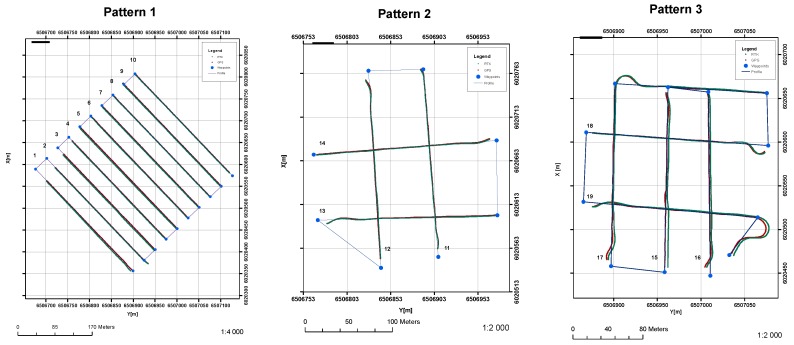
Survey pattern plans.

The experiments were divided into three phases. Phase one was data acquisition. In this phase three different patterns were planned and executed. The patterns were planned to represent typical hydrographic surveys based on the present unit (Pattern 1) (Figure 6) and the bottom object investigation plan (Patterns 2 and 3) (Figure 6). All the profiles were numbered in accordance with their execution order. Profiles 1 to 10 belong to pattern no. 1, profiles from 11 to 14 belong to pattern no. 2, and profiles from 15 to 19 belong to pattern no. 3. All data were recorded within their typical hardware configuration, meaning that no additional technical rearrangements of the unit were conducted. The unit in this configuration was prepared to undertake surveys based on best knowledge and practice including use of state-of-the-art hydrographic equipment, therefore, all equipment was calibrated, and all GNSS and INS equipment offsets were measured, and data entered into all hardware units.

Phase two of the experiment concerned logged data filtration and preparation and evaluation of navigation GPS accuracy in the dynamic measurements. To prepare for this evaluation, studies described elsewhere [1] were used.

The approach used the PL-2000 system (ETRS89/Poland CS2000 zone 6) which afforded the replacement of angular coordinates recorded by the GPS and RTK by Cartesian coordinates (in meters). This conversion (from angular GPS coordinates to Cartesian) allowed the calculations to be simplified and the results to be presented in meters. The PL-2000 coordinate system is Cartesian 2D coordinate system with northing (x) and easting (y) axes with orientations fixed to north, east, and units of measurement in meters.

### 4.1. Data Synchronization

**Figure 7:**
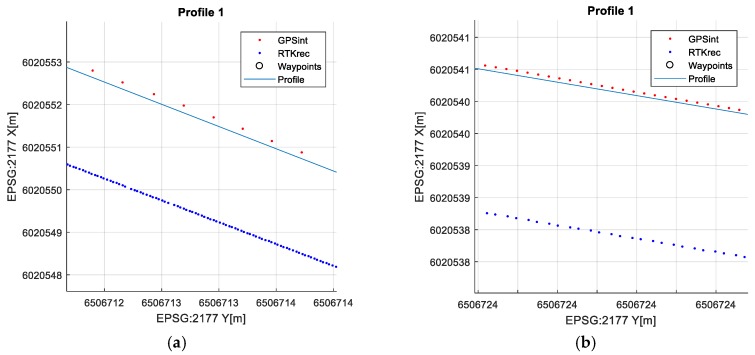
Recorded GPS and RTK tracks (**a**) different frequency (**b**) interpolated GPS positions.

Navigation GPS maximal output frequency was declared as 5 Hz, which means that the GPS position ($p_{GPS}$) was reported a maximum of five times per second (Figure 7a). To compare both the registered tracks, i.e., RTK and GPS, the data rate for both RTK and GPS should be the same (Figure 7b). However, the RTK system reported position ($p_{RTK}$) with a maximum of 50 points every second and in fact both systems registered tracks at different rates.

To align the track rates a linear interpolation was applied to the lower rate track, i.e., the GPS track. All GPS positions ($p_{GPS}$) were interpolated with the maximum number of points equal to that for the RTK ($p_{RTK}$).

Assuming a data set consisting of independent data values $x_{i}$ and dependent data values $y_{i}$, where x=1,…,n, we can find an interpolation function $\hat{y}\left(x\right)$ such that $\hat{y}\left(x_{i}\right)=y_{i}$ for every point in our data set. This means that the interpolation function goes through the given data points. Given a new x∗, we can interpolate its function value using $\hat{y}\left(x*\right)$ [43]. In the present study, a linear interpolation was used.

The estimated positions are assumed to lie on the line joining the nearest registered positions of the estimated track with $n_{G\mathrm{PS}}$ registered positions. It is assumed, without loss of generality, that the GPS coordinates transformed to PL-2000 ($X_{G}$ and $Y_{G}$) are in ascending order, then new interpolated position coordinates ${\hat{X}}_{G_{i}}$ and ${\hat{Y}}_{G_{i}}$ are calculated according to:

(9) $${\hat{X}}_{G_{i}}=X_{G_{i}}+\frac{\left(X_{G_{i+1}}-X_{G_{i}}\right)\left(n_{R}-n_{G}\right)}{\left(n_{G+1}-n_{G}\right)}$$

(10) $${\hat{Y}}_{G_{i}}=Y_{G_{i}}+\frac{\left(Y_{G_{i+1}}-Y_{G_{i}}\right)\left(n_{R}-n_{G}\right)}{\left(n_{G+1}-n_{G}\right)}$$

where: $n_{R}$ represents the RTK measurement number, that is, $n_{R}=1,\ldots ,n_{RTK}$, $n_{G}$ represents the GPS measurement number, that is, $n_{G}=1,1+r,\ldots ,n_{GPS}$, where *r* is a RTK to GPS measurement ratio calculated according to the following equation:

(11) $$r=\frac{n_{RTK}}{n_{GPS}}$$

An example of a result for the GPS position interpolation is presented in Figure 6. In every case the number of GPS measurements was increased and equals the number of RTK measurements.

### 4.2. System Offsets

The position of the unit for navigation purposes is taken from the navigation GPS placed on the top of the mast located in the geometric center of the USV. The GPS antenna location is determined by experience and typical recommendations for unmanned units; that is, the best place for navigations system is the geometric center of the unit and at the highest possible place to diminish interference with board electronics and ensure best satellite visibility. These actions were taken in the present study.

**Figure 8:**
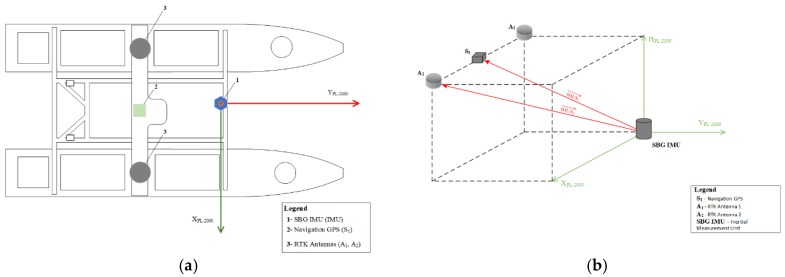
Measurement equipment location on the unit (**a**) and local unit coordinate system (**b**).

For the hydrographic equipment, this consisted of a multi beam echosounder (MBES), a precise SGB IMU, and an RTK receiver. The best practice for that equipment localization is that the IMU sensors and the MBES antenna should be as close as possible, if not, all offsets should be entered into the system. In that case, the offsets represent coordinates in the local unit coordinate system of all hydrographic equipment including the GPS, the RTK antennas, and the IMU unit, where the IMU sensor is the coordinate system origin with the Y axis parallel to the unit long axis of symmetry (Figure 8). The hydrographic equipment is calibrated, and offsets are entered into the hydrographic system in accordance with values presented in Table 4. This means that the GPS RTK position was already reported with offsets by the hydrographic system at the origin.

**Figure 9:**
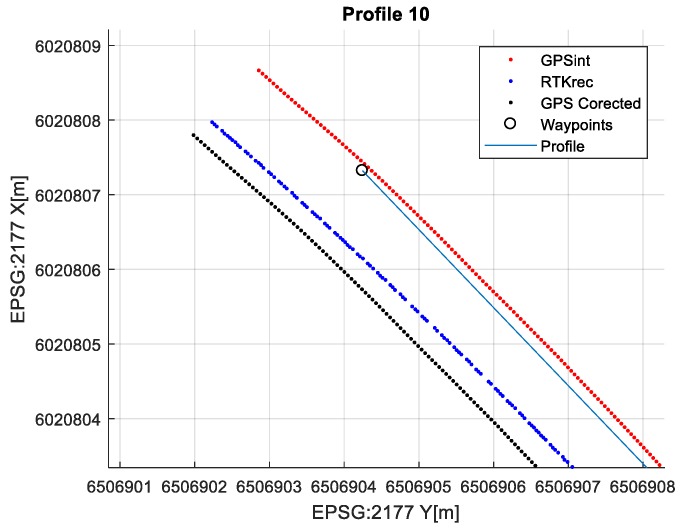
Example of corrected position for GPS at profile no. 10 (black dots).

Given that the system navigation GPS is working out of the hydrographic system and for proper evaluation a vector between the RTK and GPS readings has to be included. The corrected GPS position was calculated from already interpolated GPS positions according to the following formula:

(12) $${\hat{X}}_{G_{i}}^{*}={\hat{X}}_{G_{i}}-\left(d_{xG}\;\cos\left(HDG_{i}\right)-d_{yG}\sin(HDG_{i}\right))$$

(13) $${\hat{Y}}_{G_{i}}^{*}={\hat{Y}}_{G_{i}}-(d_{xG}\;\sin\left(HDG_{i}\right)+d_{yG}\;\cos\left(HDG_{i}\right)$$

where ${\hat{X}}_{G_{i}}^{*},{\hat{Y}}_{G_{i}}^{*}$ are the antenna coordinates of the GPS receiver in the national plane rectangular coordinate system, $d_{xG}$, $d_{yG}$ are the antenna offset values of the GPS antenna unit on the vehicle coordinate system, defined at the center of IMU(RTK) unit with the y axis being parallel to the unit symmetry axis, and the x axis being perpendicular to the y axis, and HDG is the vessel’s actual course reported by the hydrographic system (Figure 9).

### 4.3. GPS Evaluation

Having obtained all data at an equal rate, with the same coordinate system (PL-2000) and corrected the coordinates using antenna offsets, the navigation GPS accuracy may be calculated. Initially a Euclidean distance between the navigation GPS interpolated position (${\hat{p}}_{GPS}$) and the RTK referenced position was calculated, in accordance with following equation:

(14) $$d\left(p_{G\mathrm{PS}},p_{R\mathrm{TK}}\right)=\overset{n_{R\;}=n_{R\mathrm{TK}}}{\underset{n_{R}=1}{\sum }}\sqrt{{\left({\hat{p}}_{G{\mathrm{PS}}_{n_{R}}}-p_{R{\mathrm{TK}}_{n_{R}}}\right)}^{2}}$$

Consequently, the dynamic navigation GPS accuracy was calculated in accordance with formula in [44]. 

### 4.4. Cross Track Error

The cross track error (XTE) is defined as the distance between the planned sounding profile (planned USV track) and the actual unit position. The sounding profiles are represented by a line connecting two defined WPs. Figure 3 presents the XTE, which equals $l_{⊥}$. Assuming that the sounding profile is defined by two waypoints $WP_{i}\left(X_{W_{i}},Y_{W_{i}}\right)$ and the next waypoint $WP_{i+1}\left(X_{W_{i+1}},Y_{W_{i+1}}\right)$, and the actual unit position is an interpolated GPS position with coordinates ${\hat{p}}_{G{\mathrm{PS}}_{n_{R}}}\left(\;{\hat{X}}_{G_{i}},\;{\hat{Y}}_{G_{i}}\right)$ at the measurement number $n_{R}$, then the actual XTE can be calculated in accordance with the following formula:

(15) $$XTE_{n_{R}}=\frac{\left|\left(Y_{W_{i+1}}-Y_{W_{i}}\right){\hat{X}}_{G_{i}}-\left(X_{W_{i+1}}-X_{W_{i}}\right){\hat{Y}}_{G_{i}}+X_{W_{i+1}}Y_{W_{i}}-Y_{W_{i+1}}X_{W_{i}}\right|}{\sqrt{{\left(Y_{W_{i+1}}-Y_{W_{i}}\right)}^{2}+{\left(X_{W_{i+1}}-X_{W_{i}}\right)}^{2}}}$$

## 5. Results

The final results represent the calculation process outlined above. Each profile was calculated separately. Table 5 is a graphical example of the results for three representative profiles, np. 2, 6, and 10. The coordinates difference graph represents differences for the X and Y coordinates between the RTK and GPS registered positions along the selected profile. The Euclidean distance represents the results of Equation (14) along selected profiles in meters. The XTE represents the cross track error along selected profiles as a result of applying Equation (15).

Based on the above calculation, the accuracy of dynamic navigation GPS was calculated in accordance with equations described elsewhere [44,45] for each profile separately. Table 6 presents the results of the calculations for each profile. Additionally, in Table 6 the mean XTE was added to evaluate the accuracy of the trajectory tracking for each profile.

The mean values for the research study are presented in Table 7. The mean accuracy for trajectory tracking (mean unit XTE) of the USV is very satisfactory, particularly if we compare it with traditional manual profile tracking or trajectory tracking realized by a typical autopilot used for ship navigation.

## 6. Discussion

Nonlinear guidance, originally designed for UAV trajectory tracking, has been adopted and used for USV trajectory tracking. This method allows us to keep a low XTE during all sounding profiles’ tracking; more importantly, the USV course changes that keep to the track are very gentle, and do not cause significant disturbances in hydrographic measurements. The trajectory tracking and the USV response for the calculated course inputs depend on the correct PID tuning. As stated above, PID tuning was carried out during extensive field tests. The configuration employed did not show any oscillations, the course changes although robust were gentle, allowing us to keep track within specified limits for the unit to weather conditions and wind direction. The correct PID tuning is very important for course and track keeping. As mentioned elsewhere [28], the USV used in this research showed a regular oscillation for track following, caused by not having ideal PID tuning, and this can significantly affect the unit’s endurance and the quality of hydrographic measurement.

The position registered by the navigation GPS is not used directly by the USV for navigation. The pure GPS position readings are filtered using EKF and the final navigation position is calculated using all data available to the autopilot internal sensors. This technique is used widely within the robotics community to estimate a robot’s position. Consequently, the position used for navigation, as the results show, has good accuracy. The profile planned by the hydrographers, was followed based on GPS position. If we take into account the antenna position (in the center of the unit) and the offsets between the GPS antenna and the multibeam sonar (main hydrographic sensor) this causes quite significant differences between the real unit track and the sonar antenna track. The displacement differs and depends on the wind speed and direction. Practically, the profile is registered by the RTK system with offsets, therefore, from the hydrographic point of view, all data are registered correctly, however this difference can be diminished.

## 7. Conclusions

As the results show, the accuracy of trajectory tracking based on nonlinear guidance logic is suitable for hydrographic USV profile tracking, and the presented unit configuration permits very precise track following with a mean XTE of around 30 cm. This is a very good result, if we compare this performance with a traditional manned hydrographic vessel. In the authors’ opinion, trajectory tracking based on nonlinear guidance logic for hydrographic measurements can be implemented on a wide variety of USVs providing correct nonlinear guidance logic and PID parameters.

The difference between the track realized by the unit and the real sonar track can be significant and depends on wind speed and direction. This difference can be diminished using either software offsets or antenna physical displacement. The other approach is to use the RTK signal directly for navigation during the hydrographic survey.

## Figures and Tables

**Table 1:** USV technical specification.

Specification	Data
Dimensions (L × W × H)	4230 × 2080 × 1390 mm
Draft	500 mm
Weight	360 kg
Power supply	48 V 200 Ah lithium iron phosphate battery (LiFePO_4_) (16 cells) for propulsion, 24 V lead-acid battery for electronics
Endurance	12 h (cruise speed)
Motors	2 × Torqeedo Cruise 4.0
Remote control range	40 km
Telemetry data range	50 km
Payload data range	6 km
Max speed	14 knots

**Table 2:** Navigation GPS specification.

Parameter Name	Specification
Channels	72
Signal tracking:	GPS: L1C/A SBAS: L1C/A QZSS: L1C/A GLONASS: L1OF BeiDou: B1 Galileo: E1B/C2
Horizontal position accuracy ^1^	GPS and GLONAS: 2.5 m SBAS: 2.0 m
Velocity accuracy ^2^	0.05 m/s
True heading accuracy ^2^	0.3°
Operating limits	Altitude: 50,000 m Velocity: 500 m/s Acceleration: 4 g
Time to first fix	Cold start: <26 s Warm start: <1 s
Max output frequency	5 Hz

^1^ CEP, 50%, 24 h static, −130 dBm, >6 SVs; ^2^ 50% at 30 m/s.

**Table 3:** GPS RTK specification.

Parameter Name	Specification
Channels	220
Signal tracking	GPS: L1 C/A, L2E, L2C, L5 GLONASS: L1 C/A, L2 C/A, L2 P, L3 CDMA Galileo: 1 BOC, E5A, E5B, E5AltBOC Beidou B1, B2 SBAS, QZSS L-Band OmniSTAR VBS, HP, XP
Horizontal position accuracy (1 sigma)	SBAS/DGPS: 0.5 m/0.25 m PPP: 10 cm RTK: 0.8 cm + 1 ppm
Velocity accuracy	0.7 cm/s RMS
True heading accuracy	0.09 ° at 2 m baseline 0.05 ° at 1 0m baseline
Operating limits	Altitude: 18,000 m Velocity: 515 m/s Acceleration: 11 g
Time to first fix	Cold start: <45 s Warm start: <30 s
Signal reacquisition	L1/L2/L5: <2.0 s
Max output frequency	50 Hz

**Table 4:** Navigation equipment offsets.

	dy	dx	d_H_	|COGA_1_|	|COGS_1_|	Remarks
GPS	−1.22	0.16	0.89	16.402		For Equations (12) and (13)
Origin	0	0	0		15.186	
Antenna RTK 1	−1.22	−0.64	0.89			In hydrographic system
Antenna RTK 2	−1.22	0.96	0.89			In hydrographic system

**Table 5:** Graphical results for example profiles no. 2, 6, and 10.

Coordinate Differences	Euclidean Distance	XTE
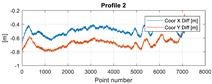	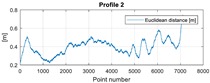	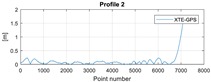
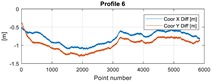	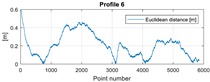	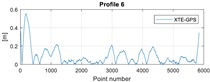
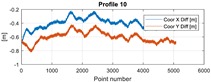	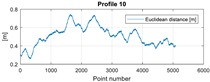	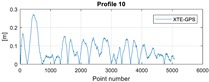

**Table 6:** Results for all profiles.

Profile No.	σ_x_	σ_y_	DRMS	2DRMS	CEP	R95	Mean XTE
1	0.4490	0.4788	0.6564	1.3129	0.5483	1.1405	0.1389
2	0.0599	0.0698	0.0920	0.1840	0.0768	0.1598	0.1080
3	0.1650	0.2108	0.2677	0.5354	0.2231	0.4640	0.0934
4	0.1029	0.0977	0.1419	0.2839	0.1182	0.2459	0.0578
5	0.1756	0.1975	0.2643	0.5286	0.2208	0.4592	0.0582
6	0.1698	0.1770	0.2453	0.4906	0.2048	0.4260	0.0881
7	0.1013	0.1447	0.1766	0.3533	0.1464	0.3046	0.0875
8	0.0857	0.0853	0.1209	0.2418	0.1009	0.2098	0.0911
9	0.0946	0.1044	0.1409	0.2817	0.1177	0.2448	0.0783
10	0.0783	0.0773	0.1101	0.2201	0.0918	0.1909	0.0750
11	0.2164	0.1547	0.2660	0.5320	0.2171	0.4516	0.2546
12	0.3149	0.0575	0.3201	0.6402	0.2120	0.4410	0.2433
13	0.1403	0.2244	0.2646	0.5293	0.2177	0.4528	0.3474
14	0.0591	0.1475	0.1589	0.3177	0.1245	0.2590	0.2315
15	0.2188	0.4595	0.5089	1.0179	0.4074	0.8475	0.3826
16	0.7284	0.2069	0.7572	1.5145	0.5362	1.1153	0.3218
17	0.5927	0.3553	0.6910	1.3821	0.5522	1.1486	1.5335
18	0.1795	0.3014	0.3508	0.7016	0.2874	0.5977	1.1249
19	0.1899	0.5650	0.5961	1.1922	0.4567	0.9499	0.4945

**Table 7:** Mean accuracy results for USV configuration.

	σ_x_	σ_y_	DRMS	2DRMS	CEP	R95	Mean Unit XTE
Mean	0.2170	0.2166	0.3226	0.6452	0.2558	0.5321	0.3058
